# Effect of Guanidinoacetic Acid Supplementation on the Performance of Calves Fed Milk Replacer

**DOI:** 10.3390/ani14192757

**Published:** 2024-09-24

**Authors:** Kathryn J. Hazlewood, Charles A. Zumbaugh, Cassandra K. Jones, Emily M. Atkinson, Hannah L. R. Tingler, Vivienne K. Inhuber, Micheal J. Brouk, Reshma M. Antony, Evan C. Titgemeyer

**Affiliations:** 1 Department of Animal Sciences and Industry, Kansas State University, Manhattan, KS 66506, USA; 2 Alzchem Trostberg GmbH, 83308 Trostberg, Germany

**Keywords:** creatine, growth, guanidinoacetic acid, steers

## Abstract

**Simple Summary:**

Creatine is a metabolite that animals use to store energy within tissues. Animals, including cattle, can synthesize creatine by first generating guanidinoacetic acid from the amino acids glycine and arginine, followed by conversion of guanidinoacetic acid to creatine. In some situations, the body’s production of creatine is not adequate to support maximal growth. We examined the supplementation of guanidinoacetic acid to calves fed milk replacer to determine if growth performance would be enhanced. Supplementation of guanidinoacetic acid tended to improve the growth of the calves, which may suggest that milk replacer alone was not sufficient to supply adequate amounts of creatine through the combination of diet and endogenous production in the body. Thus, dietary supplementation with guanidinoacetic acid may be a feasible means of improving growth in calves fed diets containing inadequate amounts of creatine.

**Abstract:**

Guanidinoacetic acid (GAA) is the direct precursor to creatine, which serves as an energy reserve mechanism in the body. We evaluated the effects of GAA supplementation on the growth performance of calves fed milk replacer. Forty-five Holstein–Angus steer calves (40.9 kg, approximately 1 week old) were assigned to 1 of 3 treatments to assess growth performance and data from 41 calves were analyzed. Treatments were provided in the milk replacer for 42 d and included 0, 1, or 2 g GAA/d. Calves were fed 2.84 L milk replacer twice daily with ad libitum access to starter feed and water. Treatments ended on day 42 prior to a 17-day weaning period. Supplementation of GAA increased (*p* < 0.01) plasma concentrations of GAA (0.42, 0.51, and 0.67 mg/L for 0, 1, and 2 g GAA/d, respectively) and creatine (18.7, 22.1, and 24.4 mg/L for 0, 1, and 2 g GAA/d, respectively). Bodyweight tended to linearly increase (*p* = 0.09) with increasing GAA on d 59 (91.2, 98.3, and 98.6 kg for 0, 1, and 2 g GAA/d, respectively). Daily gains from day 0 to 59 tended to increase with GAA provision (*p* = 0.09; 0.86, 0.97, and 0.98 kg/d for 0, 1, and 2 g GAA/d, respectively). Starter feed dry matter intake tended to linearly increase (*p* = 0.06) with GAA supplementation (0.201, 0.278, and 0.286 kg/d for 0, 1, and 2 g GAA/d, respectively). Treatments providing 1 and 2 g GAA/d affected performance similarly. No differences among treatments were observed for health (respiratory and fecal) scores during the application of treatments or during the subsequent weaning period. The inclusion of GAA in milk replacer tended to increase the gain of calves, and this was associated with elevated starter feed intake.

## 1. Introduction

Guanidinoacetic acid (GAA) is the direct precursor to creatine, which is important in regenerating adenosine triphosphate during high-intensity work [1]. Synthesis of creatine requires three amino acids in a two-step process beginning with the amidino group from arginine transferred to glycine to generate GAA, which is methylated at the amidino group by S–adenosyl–L–methionine: N–guanidinoacetate methyltransferase in the liver resulting in creatine [2]. Creatine is then released into the circulatory system for uptake via the creatine transporter into creatine-containing cells, predominantly skeletal and heart muscle [3].

Using post-ruminal infusions of GAA, we demonstrated that cattle, like monogastric species, can utilize GAA to synthesize creatine [4,5]. Dietary GAA supplementation has been shown to increase the growth performance of finishing Angus bulls [6,7,8] and finishing Jinjiang bulls [9]; this research also demonstrated that dietary GAA supplementation increases diet digestibilities, which could account for some of the growth-promoting activity. Similarly, dietary GAA has been shown to increase growth in lambs [10,11], although Zhang et al. [12] did not observe an improvement in the growth of lambs supplemented with GAA.

The use of GAA is approved by both the European Food Safety Authority (EFSA) and the United States Food and Drug Administration (FDA) for poultry and by the EFSA for poultry and swine [13,14]. However, GAA is not currently approved for use in cattle.

Young growing animals require creatine to support tissue growth [1], but there is no information available about the effects of dietary GAA supplementation on the performance of calves fed milk replacer. Our objective was to evaluate the effect of GAA supplementation on the growth performance of milk-fed calves and the subsequent growth performance during the weaning period. We hypothesized that GAA supplementation would improve the performance of calves fed milk replacer.

## 2. Materials and Methods

All procedures involving the use of animals were approved by the Kansas State University Institutional Animal Care and Use Committee.

### 2.1. Animals and Experimental Diets

A 42-d growth study was conducted using 49 Holstein (dam)–Angus (sire) crossbred steer calves (castrated male cattle) of 5 to 9 d of age at initiation of the trial (40.9 ± 3.55 kg initial body weight (BW)). Steers were obtained in one load from a calf ranch (Fullmer Calf Ranch, Syracuse, KS, USA). Prior to arrival, calves received colostrum on the farm of birth, were transported to the calf ranch at 1 to 2 d of age, were fed milk replacer for 1 to 4 d, and then were transported to Manhattan, KS 3 d before the trial was initiated. Calves were weighed on arrival and then individually housed in hutches (Calf-tel, Germantown, WI, USA) and bedded with a layer of pine wood shavings with straw added heavily on top. Calves were staged for 3 d until the initiation of the trial, during which they were fed milk replacer and introduced to starter feed, both being the same that were fed during the trial (Table 1). A health assessment was conducted by the Kansas State Veterinary Health Center, and all calves received a subcutaneous dose of florfenicol (40 mg/100 kg BW; Nuflor; Merck Animal Health, Madison, NJ, USA) 1 or 2 d before the start of the trial.

A jugular vein blood sample was collected 1 day prior to the start of the trial into a 3-mL vacutainer tube without anticoagulant (BD Vacutainer; Beckton, Dickinson, and Company, Franklin Lakes, NJ, USA) at 1 to 3 h after feeding. Blood was allowed to coagulate and then centrifuged at 1000× *g* at 4 °C for 20 min. Serum was harvested and total serum protein was measured using refractometry [15] as an assessment of passive transfer from colostrum. Deelen et al. [15] showed refractometry measurements correlated well with serum IgG concentrations. For allocation to treatment, calves were grouped into uniform blocks of 3 animals based on arrival BW and total serum protein to achieve groups of calves with similar weight and total serum protein concentrations.

Treatments included 0, 1, or 2 g/d of guanidinoacetic acid (GAA; Creamino, 96% GAA; Alzchem Trostberg GmbH, Trostberg, Germany) provided in the milk replacer. Only 45 calves were blocked and allocated to treatment due to death loss and inconsistent intake of milk replacer. Then, One calf from each block was randomly assigned to each treatment group, resulting in 15 animals per group. One calf supplemented with 2 g GAA/d died within 24 h of trial initiation and was replaced by a calf, not initially allotted to the trial, that best matched the pretrial BW and serum protein of the calf that died. Three additional calves died during the trial period, and one calf was later identified as a non-castrated male; all data from these four calves were eliminated from the data set. The four calves removed from the data set were from three different blocks. This resulted in 41 calves in the final data set with 13 calves for control and 14 calves each for the 1 and 2 g GAA/d treatments.

Calves were fed 2.84 L of milk replacer (149 g dry matter (DM)/L) twice daily at 0700 and 1800 h, with each calf using the same bottle and nipple through the entirety of the trial. Milk replacer (K-State 26/24 AM DX; Milk Specialties Global, Eden Prairie, MN, USA; Table 1) with decoquinate (26.69 mg/kg) and 0.5 g/calf probiotic (Certillus Calf LC; Church & Dwight Co., Inc, Waukesha, WI, USA) was added to warm water (43.3 °C) and mixed for 4 to 5 min for each batch. Two batches were mixed daily. Half of the daily GAA treatment amount was added directly to each bottle before the nipple was placed on the bottle and shaken. The treatment product was added to the milk replacer in its original dry form through d 10. At this point, it was determined that the product was not fully suspended in the milk replacer, which may have resulted in some unconsumed product in the bottles after feeding. From d 11 to 42, for the 2 g GAA/d treatment, 1 g of product was mixed with 2 mL of water and 1 mL 4.5 M HCl, allowed to set for 20 min, and then neutralized with 1 mL 18% wt/wt NaOH prior to addition into the bottles; for the 1 g GAA/d treatment, mixtures used one-half the amounts described. Milk replacer was fed to calves between 37.8 and 40.6 °C. Calves were fed in the same sequence at each feeding. Milk replacer refusals were usually nil, but when present, they were measured after each feeding.

Starter feed (Elite 18% Calf Starter TRT D22.7; Hubbard Feeds, Mankato, MN, USA) medicated with decoquinate (50.04 mg/kg) was fed to calves individually for ad libitum intake through the entirety of the study (Table 1). Starter was provided at the morning feeding and refusals were measured the following morning to calculate daily intake. Initially, calves received 0.045 kg, which was replaced daily for the first 10 d. After this point, a set amount of feed was determined every morning with the goal of maintaining ad libitum access. Refusals were measured and, if the leftover feed was dry and uncontaminated, new feed was added to the refusals until the set amount was reached. If the feed was wet to the touch, all feed was discarded; a composited sample from the discarded feed was taken and analyzed for DM. If little or no feed was left at the evening feeding, the full daily amount of feed was added. For instances where feed refusals were not weighed, the daily starter intake was calculated as the average from the previous and following daily intakes; this constituted 2.23% of all observations. Water was provided for ad libitum access and refreshed at each feeding.

Bottles and nipples were cleaned after each feeding. Bottles were first washed with warm water and soap and then fully submerged in a 0.016% chlorohexidine solution (Chlorhexidine Solution; Aspen Vet, Loveland, CO, USA) for 20 to 30 s and air-dried. Due to health concerns, a third step was added on d 26, with bottles briefly submerged in a 0.6% wt/vol bleach solution and air-dried. Nipples were cleaned using the same process. The milk replacer mixer was cleaned and sanitized after each feeding. It was first emptied of any remaining milk replacer, rinsed with hot water until clear, and filled with 0.016% chlorohexidine solution, which was retained for 1 to 2 min. The chlorohexidine solution was drained from the mixer and the lid was removed and air-dried. Manure and wet straw were removed from hutches daily in the evening. After the first 2 weeks, and weekly thereafter, hutches were completely cleaned out and new straw was added. 

Health was assessed twice daily at each feeding. An evaluation of each animal was based on a scale of 0–3 for respiratory, lameness, and fecal consistency using a system adapted from the University of Wisconsin-Madison School of Veterinary Medicine Calf Health Scoring Chart (https://fyi.extension.wisc.edu/heifermgmt/files/2015/02/calf_health_scoring_chart.pdf (accessed on 18 september 2024); [16]). Evaluation of respiratory health was the sum of general appearance, eye and nasal discharge, and cough scores. Adaptations included the addition of a general appearance category with 0 = energetic, normal demeanor, and comes to feed eagerly; 1 = slightly depressed and does not come to feed as readily; 2 = depressed demeanor, low energy, and does not come to feed; and 3 = depressed demeanor and sickly looking. Cough was scored as yes = 1 or no = 0. Rectal temperature and ear tilt scores were not utilized. If the respiratory health score was greater than or equal to 4, a veterinarian was consulted. If prescribed by the veterinarian, florfenicol (40 mg/100 kg BW) was injected subcutaneously and that animal was observed carefully for further respiratory symptoms; no second treatments were needed.

The electrolyte solution was given to calves with poor fecal consistency or low milk replacer intake. Calves that refused all milk replacer were given one half to one full dose (1 dose = 1.89 L) of electrolyte at the time of feeding. Calves with a fecal consistency of 3, or with a fecal consistency of 2 with reduced milk replacer intake, were given a half dose of electrolyte solution between feedings. Calves received one half to one dose of electrolyte, according to severity, until 1 d after fecal consistency and intake improved. The electrolyte was first attempted to be fed by bottle and, if refused, was tubed. Epic (Tomlyn Products, Fort Worth, TX, USA) was used from d 0 to 1, Re-sorb (Zoetis, Kalamazoo, MI, USA) was used from d 2 to d 21, and Hydra-Lyte (AgriLabs, Shenandoah, IA, USA) was used from d 21 to d 38, based on availability. No doses of electrolyte were needed after d 38.

Body weight and hip height were measured on d 0, 14, 28, and 42 for assessment of growth performance during treatment provision. On d 14, 28, and 42, a jugular vein blood sample was collected into a 10-mL vacutainer tube (BD Vacutainer; Beckton, Dickinson, and Company) containing sodium heparin starting 3.5 h after feeding. Tubes were immediately inverted multiple times and placed in ice until centrifuged at 1000× *g* at 4 °C for 20 min. Plasma was harvested into 2-mL microcentrifuge tubes and stored at −20 °C until analysis of GAA and creatine as described below.

Treatments ended after the morning feeding on d 42. Weaning was then initiated as follows. Starting at the evening feeding on d 42 through d 48, calves received 1.89 L milk replacer twice daily. From d 49 through 55, calves received 1.89 L only once daily at the morning feeding. On d 56 and following, calves received no milk replacer. Starter feed, water, and cleaning remained as previously described. Health evaluations were conducted once daily at the morning feeding. Calves were vaccinated on d 53 with Bovilis Vista 5 SQ (Merck Animal Health, Omaha, NE, USA) for infectious bovine rhinotracheitis, bovine viral diarrhea type 1 and 2, bovine respiratory syncytial virus, and bovine parainfluenza-3 and Bovilis Vision 7 with SPUR (Merck Animal Health, Omaha, NE, USA) as a clostridial vaccine including *Clostridium perfringens* Types C and D. On d 59, individual calf weight was measured.

### 2.2. Sample Collection and Analysis

Both milk replacer and starter samples were collected weekly and stored at −20 °C until analysis. Approximately 10 g of milk replacer from each sample was composited for analysis. Approximately 50 g of starter feed was composited and dried in a forced-air oven at 55 °C for 24 h and then ground through a 1-mm screen using a Thomas Wiley Mill (Thomas Scientific, Swedesboro, NJ, USA). Analyses of DM (by drying at 105 °C), organic matter [17], crude protein (N × 6.25; [18], ether extract, calcium [19], and phosphorus [18] content in milk replacer and starter feed were conducted by a commercial laboratory (SDK Laboratories; Hutchison, KS, USA). In addition, neutral detergent fiber [20], acid detergent fiber [20], and starch [21] content were analyzed in the starter feed. Liquid milk replacer was collected twice weekly and dried for 12 h in a forced-air oven at 105 °C to determine DM content. Feed samples from one week were not collected, and the resulting DM for the missing week was calculated as an average of the samples from the previous and following weeks.

Plasma was prepared for analysis of GAA and creatine by mixing equal volumes of plasma and 10% (wt/vol) sulfosalicylic acid, vortexing, freezing overnight, thawing, vortexing, centrifuging (17,000× *g*, 10 min, 4 °C), and then filtering through a 0.2-μm syringe filter. Samples were analyzed for GAA and creatine via UPLC (ACQUITY UPLC H-Class PLUS System; Waters Corporation, Milford, MA, USA) with a tunable UV detector with the wavelength set to 200 nm. The injection volume was 5 µL. The column used C18 chemistry (Acquity UPLC HSS T3 1.8 µm, 2.1 × 100 mm) and was maintained at 30 °C. The flow rate was 0.5 mL/min. The initial mobile phase was 1.01 g sodium 1-heptane sulfonic acid sodium salt, 0.9 g ammonium phosphate monobasic (NH_4_H_2_PO_4_), and 70 μL triethylamine made to 1 L with double deionized water and adjusted to pH 2.85 with approximately 0.25 mL of 7.5 M H_3_PO_4_. At 12.5 min, acetonitrile was ramped to 25% of total eluant over 2 min and then maintained at 25% for 2 min. At 16.5 min, the eluant was immediately returned to 100% of the initial mobile phase and held for 5 min. The total run time was 21.5 min. The plasma samples were held frozen for approximately 2.5 yr prior to analysis, and the possibility exists that some creatine converted to creatinine during storage. This conversion would decrease concentrations of creatine, but the effect would be expected to be proportional to the amount of creatine present; thus, proportional treatment differences in plasma creatine should be unaffected by storage time.

### 2.3. Statistical Analysis

Performance data were analyzed as a randomized block design using the mixed procedure of SAS (version 9.4; SAS Institute Inc., Cary, NC, USA) with the model including treatment as a fixed effect and block as a random effect. Orthogonal polynomial contrasts were utilized to identify linear and quadratic effects of treatment. The coefficients for the 0, 1, and 2 g/d treatments were, respectively, −1, 0, and 1 for the linear contrast and 1, −2, and 1 for the quadratic contrast. Health data were analyzed by first calculating the percentage of total health checks for which each calf received each possible score. These percentages were then analyzed as described for the performance data. The number of electrolyte doses each calf received between days 0 to 42 was analyzed as described for the performance data. Statistical significance was determined at *p* ≤ 0.05 with tendencies at 0.05 < *p* ≤ 0.15.

## 3. Results and Discussion

### 3.1. Milk Replacer

The milk replacer contained (dry matter (DM) basis) 25.3% crude protein and 23.8% ether extract (Table 1). The protein in the milk replacer was predominantly whey protein, and the ingredients providing the whey protein were expected to contain less creatine than the milk from which they were isolated. The milk replacer contained 227 mg creatine/kg DM, and no measurable amount of guanidinoacetic acid (GAA). Edison et al. [22] found bovine milk contained 72 mg creatine/L, which would translate to 554 mg/kg DM (assuming milk contains 13% DM). Thus, our milk replacer contained 41% as much creatine as bovine milk on a DM basis. Based on an average milk replacer intake of 812 g/d, 0.18 g creatine/d was provided via the milk replacer.

### 3.2. Plasma Concentrations of Guanidinoacetic Acid and Creatine

In response to providing GAA in milk replacer to calves, there were significant linear increases in plasma concentrations of GAA and creatine (Table 2). This demonstrates that (1) significant amounts of the supplemental GAA were available to the calves, and (2) calves increased endogenous production of creatine as increased amounts of GAA were available. These responses are consistent with previous observations when GAA was supplemented post-ruminally to cattle [4,5,23,24], and support observations from rats that most GAA is converted to creatine regardless of the creatine status [25]. From these observations, we conclude that (1) creatine production in calves fed milk replacer is limited by GAA availability, and (2) increased creatine production in response to GAA supplementation is a probable explanation for the improvements in calf performance in response to GAA supplementation (described in Section 3.3). It is unknown if the improved growth performance was a direct effect of GAA or creatine availability or was mediated through increases in intake of the starter feed (see Section 3.3).

Supplementation of GAA linearly increased plasma concentrations of GAA and creatine on all 3 sampling days (*p* < 0.01; Table 2), although the effects of GAA supplementation on plasma creatine were greater on day 14 than on day 42 (linear treatment × linear day, *p* = 0.02). This may reflect simple dilution of the creatine as calves grew during the study, although it could also reflect changes in GAA utilization for creatine synthesis and/or changes in the transport of creatine into tissues as calves aged. Plasma creatine concentrations decreased (*p* < 0.05) over time for calves receiving 2 g/d GAA, whereas plasma creatine of the control calves showed numeric linear increases over time. This argues against age-related changes in GAA utilization or creatine transport as the explanation for the treatment × day interaction for plasma creatine.

### 3.3. Growth Performance

Calves supplemented with GAA tended to have greater (linear, *p* = 0.14) body weight (BW) compared to control after 42 d (Table 3). After weaning (d 59), GAA-supplemented calves tended (linear, *p* = 0.09) to be ~7.25 kg heavier than the control group. There was a linear increase (*p* = 0.005) in hip height on d 14 with increasing GAA, but hip height did not differ among treatment groups after this point (*p* > 0.15; Table 3). There was a tendency for average daily gain (ADG) to be greater with GAA during treatment provision (linear, *p* = 0.15) and weaning (linear, *p* = 0.14) compared to control. Over the entire trial (d 0 to 59), ADG tended (linear, *p* = 0.09) to be 13% greater in calves supplemented GAA than in control calves.

Milk DM intake (DMI) did not differ among groups (*p* ≥ 0.21), which was expected because the milk replacer was limit fed. Starter DMI tended to be greater for calves supplemented with GAA (d 0 to 42, linear, *p* = 0.06; d 42 to 59, linear, *p* = 0.07; d 0 to 59, linear, *p* = 0.06). This resulted in tendencies for total DMI to be greater with GAA at all time points (d 0 to 42, linear, *p* = 0.10; d 42 to 59, linear, *p* = 0.07; d 0 to 59, linear, *p* = 0.07). However, the increase in feed consumption in response to GAA supplementation was proportional to increases in ADG, so there were no differences in gain/feed (*p* ≥ 0.38). Both GAA supplementation treatment groups yielded similar results during the trial.

Although GAA was only supplemented for 42 days, the positive effects on the intake of solid feed and BW gain continued between days 42 and 59. Importantly, this demonstrated that there were no negative consequences of discontinuing the GAA supplementation. The most obvious explanation for the beneficial effects of prior supplementation with GAA during the weaning phase (days 42 to 59) would be the increased feed intake. In addition to greater intakes providing more nutrients to the calves, the greater intake of starter feed would support ruminal development and ease the transition of the calves through the weaning phase [26].

The basal dietary supply of creatine from milk replacer was around 0.18 g/d. Our supplemental levels were much greater than this amount and likely provided more creatine to the calves than might be consumed by calves nursing their dams. Without any previous data to predict optimal levels of supplemental GAA, we selected amounts greater than typical intakes to generate responses that would be maximal. Because 1 and 2 g GAA/d yielded similar results throughout the trial, the optimal amount of supplemental GAA appears to be between 0 and 1 g GAA/d. It is possible that calves may not benefit from GAA supplementation if the milk replacer contains more creatine than ours. More research is needed to determine requirements more precisely in young, milk-fed calves. Because 2 g GAA/d led to greater plasma concentrations of creatine than 1 g GAA/d but did not further improve performance, our results support observations that creatine production from GAA is limited by GAA availability and not by the animal’s requirements for creatine. Ardalan et al. [4] and Speer et al. [23] observed that high levels of GAA supplementation increased urinary excretion of creatine, which eliminates excess creatine from the body; in those studies, urinary excretion of GAA remained low, even in the face of high amounts of GAA supplementation.

In ruminants, promising results have been observed with supplemental GAA. Improvement in BW has been observed in multiple studies with finishing bulls fed various amounts of GAA for 42 to 104 d [6,7,8,9]. In lambs, GAA supplementation for 70 d increased final BW and resulted in greater carcass weights with greater lean meat weight [11]. Similarly, GAA provided with ruminally protected Met increased final BW, total weight gain, and ADG for the first 60 d of a 90 d study in Tan lambs [10]. In contrast, supplementing lambs with GAA for 100 d did not alter the final BW or ADG [12]. Supplementation of GAA appears to increase growth in ruminants, but there is no direct comparison in the literature for the pre-ruminant calves used in this study.

The most obvious mechanism for the improvements in BW and ADG with GAA supplementation is increased creatine status in the calves. Although we did not measure creatine concentration in any tissue, increases in plasma creatine concentrations in response to GAA supplementation support the absorption of GAA with subsequent methylation to form creatine. Another potential mechanism for the growth-promoting effects of GAA is the sparing of arginine from use in GAA synthesis. Creatine provides feedback inhibition of arginine: glycine amidinotransferase [2], thereby decreasing arginine consumption for GAA synthesis when creatine supplies are adequate. Although recent research has demonstrated that arginine sparing by GAA supplementation may be important for gestating cows [27], arginine is not typically considered a limiting amino acid for calves fed milk replacer. Supplementation of arginine to calves fed reconstituted whole milk powder did not affect growth performance, although it did increase villus height in the duodenum and jejunum [28]. Another mechanism by which GAA may improve performance is the improvement of diet digestion. Li et al. [6] and Liu et al. [7,8] observed that dietary GAA supplementation increased diet digestibility in cattle, although Ardalan et al. [5] and Speer et al. [24] found that post-ruminal provision of GAA did not affect diet digestibility. In the current study, GAA was provided in the milk replacer by bottle and would be expected to bypass the rumen and, therefore, have limited impact on ruminal function. We observed no effect of GAA supplementation on fecal scores or electrolyte provision, suggesting that intestinal function was not strongly affected by GAA supplementation. 

Tissue-level mechanisms by which GAA improves growth in cattle have not been explicitly described. However, lambs supplemented with GAA grew faster, demonstrated increased concentrations of plasma insulin-like growth factor-1, and had greater phosphorylation of Akt and mammalian target of rapamycin in longissimus thoracis [11]. When activated, the Akt/mammalian target of rapamycin pathways promotes skeletal muscle growth [29]. Additionally, GAA supplementation to lambs decreased expression and concentration of myostatin, an inhibitor of myofibrillar growth, in longissimus thoracis [11]. How GAA alters these pathways is unknown, but they may be related to one or more of the potential mechanisms discussed above (creatine, arginine, and digestion).

As expected, milk DMI did not differ among treatments due to the limited amount provided to the calves. Calves were allowed ad libitum access to starter feed, and intake of starter feed tended to increase in this study when GAA was supplemented. Li et al. [6] observed an increase in DMI of growing bulls over 104 d, with 2 levels (0.6 and 0.9 g/kg DM) of GAA yielding similar increases in intake compared to either control or a lower level of supplementation (0.3 g GAA/kg DM). In addition, average daily feed intake tended to increase in Jinjiang bulls supplemented up to 2 g GAA/kg DM for 42 d [9]. Ultimately, the increase in starter feed DMI we observed could be due to the increase in gain because calves need more energy to support greater growth. Alternatively, increases in intake in response to GAA would result in more energy consumption with the potential to increase growth.

### 3.4. Health Outcomes

No differences (*p* > 0.05) in health scores were observed among treatments for respiratory or fecal scores during treatment provision or weaning (Table 4). Calves receiving 1 g GAA/d tended to have a greater percentage of days at a respiratory score of 4 or more (quadratic, *p* = 0.12), but these represented only 0.5% of total days. In addition, 1 g GAA/d tended (quadratic, *p* = 0.10) to have more days at a fecal score of 1 compared to other groups during the weaning period. Very little lameness occurred in our experiment and there were no significant differences in calf health scores for lameness (data not presented).

For gut health management, electrolyte was fed as described in the methods. No differences (*p* > 0.05) in electrolyte doses were found among groups during treatment provision, and no electrolyte was provided during weaning (Table 4).

Our calves had normal health incidences for conventional rearing of neonatal dairy calves [30]. Antioxidant status has been shown to be enhanced by GAA supplementation in pigs [31] and bulls [9], which might aid in immune function. However, the increased supply of GAA could consume methionine for methylation reactions [24]. Because methionine supplementation can improve bovine immune function [32], we postulate that GAA could be detrimental to the actions of the immune system. Conversely, creatine produced from GAA can benefit the immune system in various ways. Activities of CD4+ and CD8+ T cells in response to pathogens depend on the high availability of ATP, which is supported in these cells by creatine and high levels of creatine kinase B [33]. Also, creatine favors the development of M2 macrophage and decreases the expression of adhesion molecules and cytokines; both effects reduce inflammation [33]. However, health was not different among treatment groups, suggesting that immune function was neither greatly improved nor inhibited by GAA supplementation.

## 4. Conclusions

Our results demonstrate that the performance of milk-fed, dairy/beef-cross steers fed milk replacer can be improved with the supplementation of GAA in the milk replacer. The inclusion of GAA in milk replacer fed to calves tended to increase ADG, and this was associated with increased starter feed intake. It is unknown if the responses would be similar in either heifers or calves with only dairy-breed genetics. Performance responses to GAA were similar when GAA was supplemented at 1 or 2 g GAA/d, so the estimated range of the supplemental requirement can only be estimated as somewhere between 0 and 1 g GAA/d. Future work will be necessary to more precisely determine the amount of supplemental GAA needed to optimize performance. Moreover, the requirement for supplemental GAA will likely be affected by the dietary content of creatine, so the magnitude of change in performance in response to GAA supplementation will likely vary somewhat among diets. The benefits in gain and intake were maintained through the weaning period after treatments were no longer provided. Feeding GAA to cattle is not currently approved for use in all countries, and this limits the breadth of its use until such approvals are in place.

## Figures and Tables

**Table 1:** Composition of milk replacer and starter feed fed to Angus × Holstein calves.

Item (% of Dry Matter)	Milk Replacer ^1^	Starter Feed ^2^
Dry matter (% of as fed)	97.00	95.16
Organic matter	94.98	93.61
Crude protein	25.33	20.55
Neutral detergent fiber	-	16.94
Acid detergent fiber	-	10.65
Starch	-	33.4
Ether extract	23.81	3.61
Calcium	0.86	1.25
Phosphorus	0.65	0.55
Creatine	0.0227	-

^1^ Ingredients: dried whey, dried whey protein concentrate, dried whey product, animal fat and coconut oil (preserved with BHA and BHT), dried skimmed milk, dextrose, lecithin, dicalcium phosphate, calcium carbonate, L-lysine monohydrochloride, DL-methionine, hydrolyzed yeast, maltodextrins, iron proteinate, natural and artificial flavors, manganese proteinate, zinc proteinate, selenium yeast, copper proteinate, silicon dioxide, dried *Bifidobacterium longum* fermentation product, dried *Lactobacillus acidophilus* fermentation product, cobalt proteinate, ethylenediamine dihydriodide, vitamin A supplement, vitamin D_3_ supplement, vitamin E supplement, ascorbic acid, magnesium oxide, niacin supplement, calcium pantothenate, vitamin B_12_ supplement, thiamine mononitrate, riboflavin supplement, pyridoxine hydrochloride, biotin, folic acid, choline chloride, sodium silico aluminate, mono and diglycerides of edible fats or oils. ^2^ Ingredients: grain products, plant protein products, molasses products, roughage products, calcium carbonate, processed grain by-products, forage products, vegetable oil, calcium phosphate, salt, magnesium oxide, magnesium sulfate, potassium sulfate, propionic acid, ammonium hydroxide, acetic acid, sodium carboxymethylcellulose, sodium hydroxide, vitamin E supplement, selenium yeast, benzoic acid, zinc proteinate, manganese proteinate, sorbic acid, *Aspergillus oryzae* fermentation extract, vitamin A supplement, ferrous sulfate, vitamin D_3_ supplement, methylparaben, propylparaben, vitamin B_12_ supplement, riboflavin supplement, niacin supplement, thiamine mononitrate, d-calcium pantothenate, natural and artificial flavor, folic acid, biotin, copper proteinate, pyridoxine hydrochloride, ethylenediamine dihydriodide, menadione sodium bisulfite complex (source of vitamin K activity), tartaric acid, cobalt proteinate, verxite granules, choline chloride, silicon dioxide, mineral oil, natural flavor.

**Table 2:** Plasma concentrations of guanidinoacetic acid and creatine in Angus × Holstein calves fed milk replacer supplemented with guanidinoacetic acid (GAA).

	GAA, g/d ^1^				*p*-Value for GAA	
Plasma	0	1	2	SEM ^2^	Linear	Quadratic
Guanidinoacetic acid (mg/L)						
Day 14	0.44	0.49	0.65	0.057	<0.001	0.34
Day 28	0.36	0.50	0.72			
Day 42	0.47	0.55	0.64			
Creatine ^3^ (mg/L)						
Day 14	18.1	24.6	26.7	1.33	<0.01	0.57
Day 28	17.1	19.4	23.6			
Day 42	20.9	22.3	22.9			

^1^ Treatments provided in milk replacer for 42 days. ^2^ Average standard error of mean among treatments. ^3^ Day, *p* = 0.02; Linear GAA × Linear Day, *p* = 0.02.

**Table 3:** Growth performance of Angus × Holstein calves fed milk replacer supplemented with guanidinoacetic acid (GAA).

	GAA, g/d ^1^				*p*-Value	
Item	0	1	2	SEM ^2^	Linear	Quadratic
*n*	13	14	14			
Bodyweight (kg)						
Day 0	39.9	41.5	40.7	0.98	0.27	0.06
Day 42	69.3	73.8	73.8	2.46	0.14	0.40
Day 59	91.2	98.3	98.6	3.39	0.09	0.35
Hip height (cm)						
Day 0	61.2	61.8	62.3	0.84	0.17	0.97
Day 42	68.7	70.1	70.2	1.00	0.20	0.51
Day 0 to 42 gain	7.4	8.3	7.9	0.77	0.63	0.51
Average daily gain (kg/d)						
Day 0 to 42	0.69	0.77	0.79	0.049	0.15	0.58
Day 42 to 59	1.30	1.45	1.46	0.074	0.14	0.43
Day 0 to 59	0.86	0.97	0.98	0.049	0.09	0.45
Milk dry matter intake (kg/d)						
Day 0 to42	0.809	0.812	0.815	0.012	0.72	0.97
Day 42 to 59	0.374	0.374	0.374	0.0003	0.21	0.46
Days 0 to 59	0.683	0.686	0.688	0.0089	0.71	0.99
Starter dry matter intake (kg/d)						
Day 0 to 42	0.201	0.278	0.286	0.034	0.06	0.36
Day 42 to 59	1.694	1.995	2.024	0.132	0.07	0.38
Day 0 to 59	0.631	0.772	0.786	0.060	0.06	0.36
Total dry matter intake (kg/d)						
Day 0 to 42	1.011	1.090	1.101	0.041	0.10	0.47
Day 42 to 59	2.067	2.369	2.398	0.132	0.07	0.38
Day 0 to 59	1.315	1.458	1.475	0.064	0.07	0.39
Gain/feed (kg:kg)						
Day 0 to 42	0.677	0.697	0.709	0.026	0.38	0.89
Day 42 to 59	0.626	0.621	0.609	0.016	0.47	0.89
Day 0 to 59	0.653	0.661	0.663	0.012	0.55	0.83

^1^ Treatments provided in milk replacer for 42 d with weaning initiated on day 42. ^2^ Average standard error of mean among treatments.

**Table 4:** Percentage of days of calf health scores and electrolyte dosages provided to Angus × Holstein calves fed milk replacer supplemented with guanidinoacetic acid (GAA).

	GAA, g/d ^1^				*p*-Value	
Item/Period/Score	0	1	2	SEM ^2^	Linear	Quadratic
Respiratory score ^3^	------ % of days ------					
Days 0 to 42						
0	80.9	83.2	82.5	3.0	0.71	0.67
1	13.8	10.5	12.6	1.6	0.60	0.16
2	4.2	4.4	3.4	1.3	0.68	0.71
3	1.0	1.4	1.3	0.5	0.69	0.72
4+	0.1	0.5	0.2	0.2	0.77	0.12
Days 42 to 59						
0	93.8	92.1	94.6	1.9	0.73	0.30
1	5.5	7.1	4.9	1.7	0.77	0.33
2	0.5	0.8	0.4	0.5	0.88	0.49
3	-	-	-	-	-	-
4+	-	-	-	-	-	-
Fecal score ^4^						
Days 0 to 42						
0	36.6	40.4	38.7	2.7	0.58	0.40
1	40.2	38.6	40.4	1.6	0.92	0.37
2	18.0	15.3	17.0	1.9	0.70	0.33
3	5.2	5.6	3.9	1.2	0.43	0.47
Days 42 to 59						
0	63.3	56.6	58.7	3.4	0.36	0.30
1	33.5	40.4	36.4	2.6	0.44	0.10
2	3.2	2.9	4.4	2.0	0.66	0.72
3	0.0	0.0	0.4	0.2	0.25	0.49
Electrolyte	doses ^5^					
Days 0 to 42	1.9	2.4	1.7	0.7	0.81	0.50
Days 42 to 59	-	-	-	-	-	-

^1^ Treatments provided in milk replacer for 42 days and weaning initiated on day 42. ^2^ Average standard error of mean among treatments. ^3^ Evaluation of respiratory health was the sum of general appearance, eye and nasal discharge, and cough scores. See text for details. ^4^ 0 = normal, fully formed, 1 = semi-formed, pasty, 2 = loose, but stays on top of bedding, 3 = watery, sifts through bedding. ^5^ Doses of electrolyte over the period, 1 dose = 1.89 L.

## Data Availability

Data are contained within the article.
